# Survey of Japanese researchers and the public regarding the culture of human embryos *in vitro* beyond 14 days

**DOI:** 10.1016/j.stemcr.2023.02.005

**Published:** 2023-03-23

**Authors:** Hideki Yui, Kaori Muto, Yoshimi Yashiro, Saori Watanabe, Yukitaka Kiya, Kumiko Fujisawa, Kana Harada, Yusuke Inoue, Zentaro Yamagata

**Affiliations:** 1 University of Yamanashi Faculty of Medicine Graduate School of Medicine, Chuo City, Japan; 2 The University of Tokyo Institute of Medical Science, Minato-ku, Japan; 3 Tokyo Metropolitan Geriatric Hospital and Institute of Gerontology, Itabashi-ku, Japan; 4 Kanagawa University of Human Services School of Health Innovation, Kawasaki City, Japan

**Keywords:** human embryos, in vitro culture, 14-day rule, attitudes survey, Japanese researchers and the public

## Abstract

The International Society for Stem Cell Research (ISSCR) has eliminated its prohibition on research involving the culturing of human embryos beyond 14 days within the updated 2021 guidelines. We conducted a survey of Japanese researchers working in stem cell- or embryo-related research (n = 535) and the public (n = 3,000) about their attitudes toward the 14-day rule. Among the researchers, 46.2% agreed that embryos could be cultured beyond 14 days, a result that was slightly lower among the public (37.9%). Among those that disagreed with embryo culturing beyond 14 days, 9.5% of researchers and 5.1% of the public agreed with culturing embryos within 14 days. Among the public, higher comprehension levels correlated with both agreement and disagreement with the culture of embryos beyond 14 days compared with “cannot judge.” Further research and pubic discourse are necessary in order to better understand the factors informing participant decisions regarding the 14-day rule.

## Highlights

• Research activity involving human embryo culture beyond 14 days
• Survey of Japanese researchers working on stem cell- or embryo-related research
• Public’s attitudes toward research involving the culture of human embryos

## Teaser

Hideki Yui and colleagues conducted a survey of Japanese researchers and the public about their attitudes toward research activity involving human embryo cultures beyond 14 days. They presented that information dissemination for the public was necessary to raise the level of the debate even if this means an increase in both support and opposition.

## Introduction

In 2021, the International Society for Stem Cell Research (ISSCR) updated their guidelines for stem cell research and clinical translation (ISSCR, 2021). In this update, the ISSCR eliminated the widely recognized prohibition involving the culturing of human embryos beyond 14 days. ISSCR recommend that if local policies and regulations permit, and if there is public support, specialized scientific and ethics oversight processes may permit research beyond 14 days (recommendation 2.2.2.1). The 14-day rule is reflected in numerous laws, regulations, and guidelines within many countries, where human embryos must be cultured *in vitro* for no longer than 14 days. ISSCR Guidelines Committee members explained the context in which these guidelines were developed (Lovell-Badge, 2021; Lovell-Badge et al., 2021; Anthony et al., 2021).

The 14-day rule has been adopted in Japan; however, the rules for human embryo research in Japan are complex (Yui et al., 2022). Table S1 highlights government guidelines that stipulate the 14-day rule. The Guidelines for the Handling of Specified Embryos are established under the Act on the Regulation of Human Cloning Techniques, while other guidelines established by the government are not linked to any law. Human cloned embryos, mitochondrial replacement on surplus embryos, derivation of human embryonic stem cells (ESCs) from surplus embryos, genome editing on surplus embryos, and creating new embryos for assisted reproductive technology research must be undertaken adhering to separate special guidelines. When initiating such research, scientists must undergo special ethics review as specified in the guidelines listed in Table S1. These guidelines stipulate that human embryos should only be used for research activities within 14 days after fertilization or before the appearance of the primitive streak. However, research using surplus embryos outside the scope of such guidelines are conducted in accordance with the guidelines for medical research involving human subjects (Ethical Guidelines for Medical and Biological Research Involving Human Subjects, established in 2021 and last revised in 2022). These guidelines are not specific to embryo research and thus do not contain any provisions regarding the 14-day rule. It is unlikely that the ethics review required under the guidelines would allow culture beyond 14 days. In addition, guidelines for gametogenesis from induced pluripotent stem cells (iPSCs) are defined in the Guidelines on the Research on Producing Germ Cells from Human iPSCs or Human Tissue Stem Cells (established in 2010 and last revised in 2022). However, since the creation of embryos using such germ cells is prohibited, the 14-day rule does not appear in these guidelines.

There have been several public and stakeholder perception studies that touch upon embryo research, including motivations among donors of human embryos. One systematic review identified that research purpose, treatment stage, embryo quality, religious beliefs, and altruism appeared to be important factors for donation among embryo donors (Hug, 2008). In addition, several surveys of attitudes toward using human embryos in research, including ESCs, have been conducted (Hudson et al., 2005; Nisbet, 2005; Pardo and Calvo, 2008; Einsiedel et al., 2009; Evans and Kelley, 2011; Critchley et al., 2013). Regarding the extension of the 14-day rule, there were a total of 135 discussions on the topic in Chinese social media and news apps (Weibo, WeChat, and Tencent news) between May 27th and August 18, 2021, according to the Chinese big data public opinion platform Sina Yuqingtong, indicating that the overall attitude toward the debate was relatively neutral (46.21%) and supportive (31.82%) (Peng et al., 2022). However, there have been no large-scale surveys of stakeholder and public perceptions toward culturing embryos beyond 14 days in Japan or elsewhere. As the ISSCR guidelines stipulate the need for public support prior to culturing embryos beyond 14 days, it is critical to assess stakeholder and public attitudes toward embryo research that extends beyond 14 days. It is anticipated that such a policy shift would require greater discourse among academic societies and the public. Therefore, we conducted two surveys to assess the attitudes of stem cell and embryo researchers (members of the Japanese Society for Regenerative Medicine [JSRM] and those who were supported by the Japan Agency for Medical Research and Development [AMED], n = 535) and the public (n = 3,000) in Japan.

The detailed method of our surveys is described in Note S1. Further, a portion of the questionnaires translated into English (including the questions used in this manuscript) are available in Notes S2 and S3. Our surveys were conducted with the approval of the Institutional Review Board of the University of Yamanashi (approval number: CS0005).

## Results

### Respondents’ characteristics

Table 1 shows the characteristics of respondents. In the researcher group, there were 588 respondents, of whom 542 were members of the JSRM. We excluded 53 respondents, who did not engage in research activity, from the analysis. A total of 535 respondents met our inclusion criteria, and their responses were analyzed. Several members of the JSRM were conducting research with support from AMED, but we were unable to determine whether those from the AMED group who did not respond to the survey link were members of JSRM. Therefore, the exact response rate of researchers was unclear. However, among JSRM members, 8.96% responded to the survey.

**Table 1:** Respondents’ characteristics

	Researchers (n = 535)			The public (n = 3,000)	
	n	%		n	%
**Age**					

20–29	21	3.9		475	15.8
30–39	104	19.4		546	18.2
40–49	172	32.1		715	23.8
50–59	161	30.1		655	21.8
60–69	66	12.3		609	20.3
70 and over	11	2.1		–	–

**Sex**					

Male	413	77.2		1,517	50.6
Female	122	22.8		1,483	49.4

**Religion**					

Non-religious	–		–	1,998	66.6
Buddhism	–		–	674	22.5
Christianity	–		–	49	1.6
Shinto	–		–	74	2.5
Islam	–		–	3	0.1
Other	–		–	8	0.3
I don’t want to answer	–		–	194	6.5

**Comprehension scores**					

0–3	–		–	262	8.7
4–6	–		–	686	22.9
7–9	–		–	1,548	51.6
10–12	–		–	504	16.8

Among those participating in the public survey, the age and sex ratios in the public group were consistent with the population distribution in Japan. Researchers were mostly in their 40s–50s, with fewer in their 20s, and more were male. Most participants reported as non-religious, followed by Buddhism, and a few reported non-Buddhist religions. The majority of participants (51.6%) had comprehension scores of 7–9 points, followed by 22.9% having a 4–6 comprehension score.

### Attitudes toward the 14-day rule

When queried on the permissibility of culturing human embryos beyond 14 days under Japanese law and guidelines, 46.2% of researchers reported agreeing that human embryos should be cultured beyond 14 days, 29.3% reported being unable to judge, and 24.5% disagreed with the culturing of embryos beyond 14 days (Table 2). In comparison, 42.9% of the public reported that they cannot judge whether embryos should be cultured beyond 14 days, 37.9% agreed that human embryos should be cultured beyond 14 days, and 19.2% reported disagreeing with the culturing of embryos beyond 14 days. Significant differences were found in all responses between the two groups (p < 0.01).

**Table 2:** Attitudes toward research activities involving human embryo cultures

Beyond 14 days				
	Researchers (n = 535)		The public (n = 3,000)	
	n	%	n	%
Agree^a^	247	46.2	1,137	37.9
Cannot judge^a^	157	29.3	1,287	42.9
Disagree^a^	131	24.5	576	19.2

Within 14 days				
	Researchers who disagreed with beyond 14 days (n = 131)		The public who disagreed with beyond 14 days (n = 576)	
	n	%	n	%
Agree^a^	51	38.9	152	26.4
Cannot judge	29	22.1	124	21.5
Disagree^a^	51	38.9	300	52.1

a p < 0.01. The chi-squared tests were performed for comparison between researchers and the public. p values were adjusted with the Bonferroni correction.

Among participants in both groups who disagreed with culturing beyond 14 days, we asked about their level of agreement of culturing embryos within the 14-day limit. Of the 131 researchers who disagreed with culturing embryos beyond 14 days, 38.9% reported agreeing to culturing embryos within 14 days (9.5% of all researchers), and the same amount of researchers disagreed with culturing embryos within the 14-day limit (Table 2). Of the 576 members of the public who disagreed about embryo culturing beyond 14 days, 52.1% disagreed with culturing embryos within the 14-day limit (10% of all public participants), 26.4% (5.1% of all public participants) agreed to culturing within 14 days, and 21.5% (4.1% of all public participants) reported that they are unable to judge. Significant differences were found in “agree” and “disagree” between the two groups (p < 0.01 in both).

Researchers who agreed to culturing embryos beyond 14 days were asked whether they would consider conducting this research activity if the 14-day rule was abolished in Japan. Among the 247 researchers, 21.9% reported that they would consider conducting research where embryos were cultured beyond 14 days (10.1% of all researchers) (Table S2).

### Relationship between research activities involving beyond/within 14 days of human embryo culture and comprehension level and religion in the public

#### Human embryo culture beyond 14 days

Members of the public with higher comprehension scores tended to agree that embryos should be cultured beyond 14 days when compared with those who reported being unable to judge; the difference was significant. For every one level increase in the comprehension score, the proportion of respondents who agreed increased (odds ratio [OR]: 2.62, 95% confidence interval [CI]: 2.34–2.94, p < 0.01) (Table 3). There was no significant difference among the groups with varying religious beliefs.

**Table 3:** Relationship between research activities involving human embryos culture beyond 14 days and comprehension score and religious belief in the public

n		Crude			Adjusted^a^		
		OR	95% CI	p value	OR	95% CI	p value
**Agree (reference: cannot judge)**							

Comprehension score^b^	2,424	2.48	2.22–2.77	<0.01	2.62	2.34–2.94	<0.01
Religion^c^ (religious beliefs) reference: non-religious	2,263	1.14	0.95–1.36	0.17	1.17	0.97–1.41	0.11

**Disagree (reference: cannot judge)**							

Comprehension score	1,863	2.18	1.91–2.49	<0.01	2.18	1.91–2.50	<0.01
Religion (religious beliefs) reference: non-religious	1,719	1.18	0.95–1.48	0.14	1.14	0.91–1.43	0.25

**Agree (reference: disagree)**							

Comprehension score	1,713	1.14	0.99–1.30	0.06	1.20	1.04–1.38	0.01
Religion (religious beliefs) reference: non-religious	1,630	0.96	0.77–1.20	0.72	1.02	0.81–1.29	0.85

OR, odds ratio. 95% CI, 95% confidence interval.

a Adjusted by age and sex.

b Comprehension score was treated as 4 continuous scales, respectively, as shown in Table 1.

c Those who answered “I don't want to answer” to the religion were excluded from the analysis.

However, public members with higher comprehension scores also tended to disagree that embryos should be cultured beyond 14 days when compared with those who reported being unable to judge. For every one level increase in the comprehension score, the proportion of respondents who disagreed increased (OR: 2.18, 95% CI: 1.91–2.50, p < 0.01). There was no significant difference among the groups with varying religious orientations.

In the comparison between “agree” and “disagree,” those with a higher comprehension score tended to “agree.” For every one level increase in the comprehension score, the proportion of respondents who answered “agree” increased (OR: 1.20, 95% CI: 1.04–1.38, p = 0.01). There was no significant difference among the groups with varying religious orientations.

#### Human embryo culture within 14 days

Members of the public with higher comprehension scores tended to agree that the embryos should be cultured within 14 days when compared with those who reported being unable to judge; the difference was significant. For every one level increase in the comprehension score, the proportion of respondents who agreed increased (OR: 2.73, 95% CI: 1.86–4.01, p < 0.01) (Table S3). There was no significant difference among the groups with varying religious beliefs.

Public members with religious beliefs were more likely to disagree than those who were non-religious when compared with those who were unable to judge (OR: 2.44, 95% CI: 1.44–4.11, p < 0.01). There was no significant difference in the comprehension scores.

In the comparison between “agree” and “disagree,” those with a higher comprehension score tended to “agree.” For every one level increase in the comprehension score, the proportion of respondents who answered “agree” increased (OR: 2.89, 95% CI: 2.06–4.03, p < 0.01). In addition, those with religious beliefs tended not to agree when compared with those who were non-religious (OR: 0.63, 95% CI: 0.40–0.97, p = 0.04). In other words, non-religious public members were more likely to agree than those with religious beliefs.

### Comparison of comprehension score among the public

We grouped the public based on their attitudes toward research involving human embryos. Table 4 presents the median and quartiles of the comprehension scores for each of the groups. [3-1] “disagree with beyond 14 days-agree with within 14 days” had the largest median and 25^th^ and 75^th^ percentile values, and [2] “cannot judge beyond 14 days” had the smallest. [1] “agree with beyond 14 days,” [3-2] “disagree with beyond 14 days-cannot judge within 14 days” and [3-3] “disagree with beyond 14 days-disagree with within 14 days” had the same median and 75^th^ percentile value; the 25^th^ percentile value of [1] was higher than that of [3-2] and [3-3].

**Table 4:** Median and 25^th^ and 75^th^ percentiles of comprehension scores in each group and comparison between groups

	Median	25th and 75th percentiles	Comparison between groups^a^
[1] Agree with beyond 14 days	8	7 and 9	[1] and [2]^b^ [1] and [3-1]^b^ [1] and [3-2] [1] and [3-3]^b^
[2] Cannot judge beyond 14 days	7	4 and 8	[2] and [3-1]^b^ [2] and [3-2]^b^ [2] and [3-3]^b^
[3-1] Disagree with beyond 14 days-agree with within 14 days	9	8 and 10	[3-1] and [3-2]^b^ [3-1] and [3-3]^b^
[3-2] Disagree with beyond 14 days-cannot judge within 14 days	8	6 and 9	[3-2] and [3-3]
[3-3] Disagree with beyond 14 days-disagree with within 14 days	8	6 and 9	–

a The Kruskal-Wallis test was performed and confirmed significant differences. The Dunn’s test was then performed for comparison between groups, and p values were adjusted by the Bonferroni correction.

b p < 0.01.

The score of [3-2] was the highest and that of [2] was the lowest, with significant differences when compared with all the other groups.

## Discussion

### Analysis of results

Public perceptions of culturing embryos were somewhat similar to those of researchers, where the latter group were more agreeable to embryo culture beyond 14 days. At an initial glance, this observation may be due to researchers having greater insight on the value of conducting embryo research past 14 days, but nearly a quarter of the researchers still disagreed, and nearly a third were unable to judge the appropriateness of permitting embryo culture beyond 14 days. Our results also demonstrated that greater comprehension of stem cell and embryo research among members of the public indicated a greater likelihood of agreement with culturing embryos past day 14. However, even those who understood the content tended to disagree with the culture beyond 14 days. Therefore, a high level of comprehension does not lead to agreement with the culture beyond 14 days. This is exemplified by the fact that the highest comprehension scores were observed for participants who disagreed with the culture beyond 14 days while agreeing with the culture within 14 days. The percentage of such respondents was higher among researchers than among the public. These respondents could have found some valid reason for supporting the culture for the 14-day period. Therefore, even though the level of the public’s comprehension can increase both the number of people in favor of and against it, it is important to provide knowledge for a sounder discussion.

Knowledge plays an important role in attitude determination. However, the simple deficit model, which posits that providing individuals with information will enhance their support for science and technology because a lack of knowledge is the root cause of their lack of support, has been met with criticism in the field of science communication (Stilgoe et al., 2014; Bauer, 2016). Attitude surveys on issues comparable to that in this study have revealed rejections of the deficit model. According to a survey conducted in Italy, a high level of scientific knowledge did not necessarily result in a favorable attitude among the public toward biotechnology, such as genetic engineering of crops, the introduction of human genes into animals for organ transplants, research on human embryos, and reproductive cloning (Bucchi and Neresini, 2002). In addition, a survey of the Japanese public’s and patients’ attitudes toward germline genome editing showed that those with a higher level of comprehension tended to be more accepting of genome editing but were also more concerned about its risks (Uchiyama et al., 2018). These arguments are supported by our results.

The public was more likely to respond “cannot judge” to the culture beyond 14 days. This was not limited to research using human embryos but is characteristic of the Japanese attitude toward science and technology more generally. For example, in an international comparison of public attitudes toward whether regenerative medicine research should be promoted (Japan, South Korea, the US, the UK, Germany, and France), Japan had the highest percentage of respondents who answered “I don’t know” (Shineha et al., 2022). Taking into account that the researchers were also Japanese, the fact that 29.3% of them answered “cannot judge” for culture beyond 14 days suggests that Japanese people may not want to cast a value judgment even if they have considerable knowledge and expertise in the subject area.

Inoue et al. surveyed the Japanese public and researchers about human-animal chimeric embryos for organ transplantation in 2012 and 2015 (Inoue et al., 2016). Their survey is comparable to the survey in this study because they surveyed members of the JSRM, which was the common population with this study; in addition, the answer choices were similar to those in this study. Among the researchers, 40.5% in 2012 (29.8% in 2015) responded “acceptable” and 31.3% (25.5%) responded “conditionally acceptable” to the creation of human-animal chimeric embryos, while 46.2% of researchers in our survey agree with culturing human embryos past day 14. Among the public, 7.5% in 2012 (6.4% in 2015) responded “acceptable” and 17.9% (16.2%) responded “conditionally acceptable” to the creation of human-animal chimeric embryos, while 37.9% of the public members in our survey agreed to culturing human embryos beyond 14 days. The comparison suggested that fewer respondents supported the creation of human-animal chimeric embryos than human embryo culturing beyond 14 days, among the public members. In addition, the higher acceptance of research activities by researchers than by the general public in Inoue et al.’s study was consistent with that in this study. The percentage of “cannot judge” was clearly higher for embryo cultures beyond 14 days for both researchers and the general public in this study than “undecided” for human-animal chimera in the Inoue et al. study. It was suggested that the intuitive value judgment for culturing human embryos beyond 14 days was more difficult than that for creating chimeric embryos and that the public intuitively fears chimeric animals. One possible context for this instinct is the potential for human-animal chimeras to create confusion in our current relationships with non-human animals and in our future relationships with such chimeras, as argued by Robert and Baylis (2003).

Religious beliefs did not affect the attitude toward culture beyond 14 days. This could be attributed to the situation in Japan, where the leaders of Buddhism, which constitutes the majority of religious belief, have not taken a particularly clear position on human embryo research or abortion. A survey of the Japanese public’s attitude toward the process of creating germ cells from iPSCs and using them to create embryos also found no relationship between religious beliefs and attitude (Sawai et al., 2021). In interpreting these results, it is necessary to bear in mind that most Japanese people are not avidly religious. There are many Japanese people whose association with Buddhism is limited to special circumstances, i.e., calling a priest at funerals, and some Japanese people may identify as either “Buddhist” or “non-religious.” Therefore, a more definitive result could have been obtained if the study was based on the strength of faith or religiosity. In addition, in the Japanese context, even if a person self-identifies as non-religious, there are many cases where there is no strong belief but merely a lack of interest and so a simple comparison with the non-religious stance in the Western world is not possible. Since 95% of the respondents were either non-religious or Buddhist, identifying differences based on religiosity would not be possible as it would require a larger sample size.

Considering the diversity in responses from our survey among members of the public and researchers, greater dialogue is needed not only between researchers and the public but also among researchers. Our findings serve as a preliminary set of results about public and stakeholder attitudes toward the 14-day rule. Yet, further research is needed to deepen the factors behind the various decisions or why some participants feel they cannot make a decision on the permissibility of culturing embryos past day 14.

### Limitations of the study

Several limitations of our study are worth noting. Firstly, there could be sampling bias in recruitment of participants who are technologically savvy and would participate in web-based surveys. Therefore, we did not include those who were over 69 years in the survey for the public. Moreover, very few young researchers responded to the survey, and the response rate of researchers was low. In addition, it remains unclear how respondents’ attitudes may have been impacted by the explanations and information provided in the videos. We did not verify, through cognitive interviews or other means, how participants understood the content of the questions. Furthermore, another study from our group indicates that the explanatory video used in this survey improved public understanding; however, that manuscript has not yet passed peer review. Therefore, it cannot be ruled out that the information provided could have biased participant responses. However, it is important to note that the large number of “cannot judge” responses from the general public is consistent with the characteristics of Japanese public attitudes toward science and technology, a finding that can contribute to policymaking.

### Conclusion

This study is one of the first large-scale surveys of researcher and public attitudes toward research activities involving the culture of human embryos beyond 14 days and serves as a reference for future policy decisions. It is anticipated that there will be policy debate regarding the length of time that embryos can be cultured in the near future. This survey indicates that not many people disagree with the elimination of the 14-day rule. However, those who do agree are less than 50% among both researchers and the public, at least in the Japanese context. It is unlikely that many members of the public will be able to judge the pros and cons of such a debate. To raise the level of the debate, information dissemination will be necessary so that the public is well informed of the pros and cons of expanding or eliminating the 14-day rule. It is imperative that future research and dialogue be conducted among key stakeholder groups and the public to help inform policy decisions.

## Experimental procedures

### Resource availability

#### Corresponding author

Further information and requests for resources should be directed to and will be fulfilled by the corresponding author, Hideki Yui (hyui@yamanashi.ac.jp).

#### Materials availability

This study did not generate new unique reagents.

#### Data and code availability

A minimal dataset of the researchers is accessible through https://figshare.com/articles/dataset/Dataset_the_researchers_/20417946.
A minimal dataset of the public is accessible through https://figshare.com/articles/dataset/Dataset_the_public_/20417907.

### Statistical analysis

Chi-squared tests were performed to compare the responses of the researchers and the public; p values were adjusted using the Bonferroni correction (Table 2). Multinominal logistic regression analyses were performed to examine the association of the attitudes toward research involving human embryo cultures and comprehension level and religion in the public (Tables 3 and S3). The Kruskal-Wallis test followed by the Dunn’s test were used to compare the comprehension scores of the groups of the public classified by different attitudes toward embryo research; p values were adjusted using the Bonferroni correction (Table 4).

The significance level was set to 0.05 (5%) in each analysis. Data were analyzed using IBM-Statistical Package for the Social Sciences (v.27). Detail information is available in Note S1.

## Author contributions

Conceptualization, H.Y., K.M., and Z.Y.; data curation, H.Y., S.W., and K.F.; formal analysis, H.Y.; investigation, H.Y., K.M., Y.Y., S.W., Y.K., K.F., and Z.Y.; methodology, H.Y., K.M., and Z.Y.; writing – original draft, H.Y.; writing – review & editing, K.M., Y.Y., S.W., Y.K., K.F., K.H., Y.I., and Z.Y.; project administration, K.M.; funding acquisition, Y.Y. and Z.Y.; validation, K.H., Y.I., and Z.Y.; supervision, Z.Y.

## Supplemental information

Document S1. Notes S1–S3 and Tables S1–S3

Document S2. Article plus supplemental information
