# Supplementary material for: Survey of Japanese researchers and the public regarding the culture of human embryos in vitro beyond 14 days
Source: Stem Cell Reports. 2023 Mar 23;18(4):799–806. doi: 10.1016/j.stemcr.2023.02.005 (PMC10147549; doi:10.1016/j.stemcr.2023.02.005)
Supplement: Document S1. Notes S1–S3 and Tables S1–S3 [file mmc1.pdf]

Stem Cell Reports, Volume *18*

Supplemental Information

Survey of Japanese researchers and the public regarding the culture of human embryos *in vitro* beyond 14 days

Hideki Yui, Kaori Muto, Yoshimi Yashiro, Saori Watanabe, Yukitaka Kiya, Kumiko Fujisawa, Kana Harada, Yusuke Inoue, and Zentaro Yamagata

Note S1. Method of the surveys

1 Research participants and data collection

We conducted two web-based attitude surveys involving a specific group of researchers and the public. As web-based surveys, informed consent was obtained from all participants by requesting them to click on a checkbox within the online platform.

1.1 Researchers

A link to the survey screen was sent to all members of the Japanese Society for Regenerative Medicine (JSRM) via email and mail at the same time. A follow-up email was sent after two weeks. In total, two emails and one mail were sent by JSRM. The same link was included in all correspondence to participants. JSRM is a national scientific society with 6052 members, most of whom are biomedical researchers working in stem cells and regenerative medicine. Researchers who are members of JSRM are likely to constitute the bulk of Japanese biomedical researchers and thus serves as a convenience sample of biomedical researchers knowledgeable in regenerative medicine research. The Japan Agency for Medical Research and Development (AMED) is a government funding agency dedicated to funding ~~bio~~medical research. A total of 345 researchers, who were supported by the Project for Regenerative/Cellular Medicine and Gene Therapies were emailed. Follow-up emails were sent after two and three weeks. In total, three emails were sent by AMED. The survey remained open from March 2 to 31, 2022.

JSRM members included non-researchers such as the journalists. We excluded those who answered that they did not engage in research activities, from the analysis because our goal was to analyze the views of biomedical researchers engaged in stem cell and regenerative medicine research.

1.2 The public

To survey the public, participants were recruited from those who had voluntarily registered with a panel of the Nippon Research Center (https://www.nrc.co.jp/english/index.html). Nippon Research Center is a private company that conducts marketing and public opinion studies on a contractual basis. We commissioned the Nippon Research Center to recruit participants, host the online survey, and collect participant responses. Adults (aged 20−69 years) were recruited randomly until the target number of 3000 respondents was reached. At the time of the survey, the age of adulthood in Japan was 20 years old. According to the Japan Research Center (in response to our inquiry), the age distribution of the panel members was as follows: 7.4% were 15−19 years old, 19.5% were in their 20s, 21.9% in their 30s, 22.2% in their 40s, 18.9% in their 50s, 7.7% in their 60s, 2.1% in their 70s, 0.3 % in their 80s, and 0.02% in their 90s. Among Japan's

population of those over 14 years old in 2022, 14.2% were in their 70s, 8.4% were in their 80s, and 2.2% were in their 90s (Statistics Bureau of Japan, 2023). The proportion of those over the age of 69 enrolled in the panel was small compared to the distribution of the overall Japanese population. One reason for the gap was that most members of the panel regularly used the internet, unlike the majority of the elderly population. Therefore, there was a potential for bias as those over the age of 69 enrolled in the panel were a distinct group within their generation. Thus, the upper age limit for respondents was set at 69 years-old. Data collection was conducted from January 5 to 13, 2022 until the number of participants reached 3,000.

2 Survey items

The survey asked for opinions on several stem cell- and embryo-related research activities and was part of a larger survey study which captured stakeholder and public views on creating embryo using gamete generated from iPSCs, using such embryos for pregnancy, creating embryo models, creating a human-pig chimera, using organs from human-pig chimeras for transplantation into a patient, and transferring an embryo after mitochondrial replacement therapy into a woman for pregnancy, and using human fetal tissue for medical research. The surveys were conducted in Japanese. The questionnaires were developed by our research team, which included experts in stem cell science and prior experience in conducting perception surveys. Survey questions were shown on individual screens and once a participant responded to the question, they were unable to return to the previous screen. Nippon Research Center's online survey platform was used to host both web-based surveys. The questions used in this study were primarily related to research activities involving the culture of human embryos beyond 14 days. Background information on these research activities, including the status of domestic regulations, were also presented.

2.1 Questions for the researchers

Attitudes about the culture of human embryos beyond 14 days were phrased as "Should research in which human embryos are cultured beyond 14 days be allowed under Japanese law and guidelines, when the embryos are not used for pregnancy?" The potential fixed responses included: "should be allowed," "cannot judge," and "should be prohibited." For respondents who answered that embryo culture beyond 14 days "should be prohibited," a subsequent question was posed: "Should embryo culture that is within 14 days also be prohibited?" and were permitted similar response categories ("should be allowed," "cannot judge," "should be prohibited"). For respondents who answered "should be allowed" were asked about a follow-up question whether they would consider conducting embryo research beyond 14 days if the 14-day rule was abolished in Japan. The responses included: "would consider," "no plan to consider," and "don't know."

2.2 Questions for the public

Members of the public were asked similar sets of questions to researchers. An initial question, "Do you think that research using fertilized ova (embryos) that are cultured beyond 14 days should be allowed in Japan?" with potential fixed responses included: "should be allowed," "cannot judge," and "should be prohibited." For those respondents who answered "should be prohibited," a follow-up question ("Do you think that the culture of human fertilized ova (embryos) within 14 days should be allowed?") was posed with the same fixed response categories.

We used a newly developed video to provide participants a backgrounder on embryo research (video available from: https://figshare.com/articles/media/Explanation_by_video/19977308). The video comprised two separate sections with eight parts. The usefulness of the video at improving the public's understanding of embryo research was confirmed in a separate study (manuscript currently under review). We scored content comprehension based on six correct/incorrect questions regarding the video contents (see Note S3) and subjective comprehension of the videos. For each correct answer, one point was added. To rate their subjective comprehension of the video, we asked respondents "How well did you understand the contents of video?" The answer choices were "I understood the contents" (three points), "I understood the contents to some extent" (two points), "I did not understand the contents very well" (one point) and "I did not understand the contents" (zero points). Subjective comprehension was evaluated for each two parts of the video. The maximum comprehension score was 12 points. In this point system, a higher score indicated a better understanding.

3 Analysis method

A simple tabulation of the results of attitudes from researchers and the public toward the culture of human embryos beyond/within 14 days was presented. Chi-square tests were performed to compare the responses of the researchers and the public; P-values were adjusted using the Bonferroni correction.

We examined the association of the attitudes toward research involving human embryos and comprehension level in the public. We performed a multinomial logistic regression analysis with attitudes toward embryo culture beyond/within 14 days as the dependent variable, while adjusting for age and sex as covariates. Additionally, to consider the influence of religion, the same analysis was undertaken between two groups: religious beliefs and non-religious. Age and comprehension scores were considered on 5 and 4 continuous scales, respectively (Table 1).

We grouped the respondents in the public based on their attitudes toward research using human embryos, and compared their comprehension scores. The grouping was as follows:

[1] “agree with beyond 14 days,” [2] “cannot judge beyond 14 days,” [3-1] “disagree with beyond 14 days – agree with within 14 days,” [3-2] “disagree with beyond 14 days – cannot judge within 14 days,” and [3-3] “disagree with beyond 14 days – disagree with within 14 days.” Median and quartile of comprehension scores for each group were calculated. Kruskal-Wallis test followed by a Dunn’s test was used for comparison between groups; P values were adjusted using Bonferroni correction.

The significance level was set to 0.05 (5%) in each analysis. Data were analyzed using IBM-Statistical Package for the Social Sciences (version 27).

Reference

Statistics Bureau of Japan. (2022). Population Estimates: January 2023 Report (in Japanese). https://www.stat.go.jp/data/jinsui/pdf/202301.pdf.

Note S2. Questionary for the researchers

Survey of Attitudes in Researchers About Stem Cell or Embryo-Related Research

Request for responses to our web-based survey

In May 2021, the International Society for Stem Cell Research (ISSCR) published new guidelines. The guidelines specify what research is acceptable and what is not acceptable in stem cell- and embryo-related research.
The purpose of this survey is to gather the opinions of researchers conducting research on stem cells and regenerative medicine in order to consider the future of Japanese laws, regulations, and guidelines for related research. In this survey, we ask for your opinions on several research activities (e.g., research in which human embryos are cultured beyond 14 days, research to create embryo models, etc.) in light of the ISSCR guidelines and Japanese laws, regulations, and guidelines.

We invite you this survey through the Japan Society for Regenerative Medicine (JSRM) and Japan Agency for Medical Research and Development (AMED). Therefore, some respondents may have received duplicate invitations to this survey. The survey can be accessed via the QR code on the mail from JSRM or the URL on the e-mail sent by JSRM or AMED. You may access the survey from any location, but we ask you to answer the survey only once per person.

1. The purpose of the web-based survey
We ask for the public's thoughts on ethical and social issues regarding regenerative medicine and stem cell research.

2. Target audience for web-based survey
Members of JSRM. Researchers conducting research related to stem cells or embryos supported by AMED.

3. Use of results, protection of personal information
Your answers will be compiled into a statistical number, such as "XXX is the percentage of respondents who answered 'XXX'". Your name and personal information will not be disclosed. Your personal information will not be handled by the University of Tokyo, University of Yamanashi, Tokyo Metropolitan Geriatric Hospital and Institute of Gerontology which are the survey

administrators. The results will never be used for any purpose other than research purposes.

Please read the above text carefully and select whether you are willing to participate in this survey or not.

1. Yes
2. No

[F] What is your age? [select one]

1. 0 – 19 years
2. 20 – 29 years
3. 30 – 39 years
4. 40 – 49 years
5. 50 – 59 years
6. 60 – 69 years
7. 70 years and above

[F] What is your sex? [select one]

1. Female
2. Male

[F] Did you access this survey screen via email or mail from JSRM or E from AMED?

1. E-mail or mail from JSRM

2. E-mail from AMED

[F] For those who accessed via e-mail from AMED. Are you a member of JSRM?

1. Yes

2. No

[Q] Should research in which human embryos are cultured beyond 14 days be allowed under Japanese law and guidelines, when the embryos are not used for pregnancy?

The ISSCR Guidelines (until the 2016 edition) prohibited the culture of embryos beyond 14 days, but the 2021 revision removed this prohibition. The 2021 guidelines state that the culture of embryos beyond 14 days may be performed after specialized ethical review.
In Japan, the relevant guidelines (*) prohibit culture beyond 14 days, on the grounds that the

primitive streak appears, and body organs begin to form by this time.

1. It should be allowed
2. It should be prohibited
3. I cannot judge

(*) Guidelines for the Handling of Specified Embryos, Guidelines on the Derivation of Human ESCs, Ethical Guidelines for Research that Involves the Use of Technology to Modify Genetic Information in Human Embryos, and Ethical Guidelines for Assisted Reproductive Technology Research that Involves the Generation of Human Embryos.

[Q] For those who answered “It should be prohibited” in embryo culture beyond 14 days. Should the embryo culture that is within 14 days also be prohibited?

1. It should be allowed
2. It should be prohibited
3. I cannot judge

[Q] For those who answered “It should be allowed” in embryo culture beyond 14 days. If Japanese laws, regulations, and guidelines were to allow this research in the future, would you consider conducting this research?

1. I would consider.
2. I have no plan to consider.
3. I don’t know.

Note S3. Questionary for the public

Survey of Public Attitudes About Stem Cell or Embryo-Related Research

[SC] What is your sex? [select one]

1. Female
2. Male

[SC] What is your age? [select one]

1. 0 – 19 years
2. 20 – 24 years
3. 25 – 29 years
4. 30 – 34 years
5. 35 – 39 years
6. 40 – 44 years
7. 45 – 49 years
8. 50 –- 54 years
9. 55 – 59 years
10. 60 – 64 years
11. 65 – 69 years
12. 70 years and above

Request for responses to our web-based survey

In recent years, the state of medical research involving human subjects has been changing rapidly, with particularly remarkable progress in regenerative medicine and stem cell research.

Regenerative medicine" is a medical treatment that aims to artificially manipulate the regenerative ability of tissues to restore impaired tissues and organs to their normal state. Experiments are being conducted to process human cells and fertilized ovum (embryo) in order to realize regenerative medicine. However, because of various bioethical concerns, there is an ongoing effort to promote international alignment on what types of experiments are acceptable. In addition to researchers, the opinions of patients, their families, and the general public are also important. This survey asks about the public's awareness of the state of regulation of research on human stem cells and fertilized eggs (embryos). We apologize for the inconvenience this may

cause you, but we appreciate your cooperation.

1. The purpose of the web-based survey
We ask for the public's thoughts on ethical and social issues regarding regenerative medicine and stem cell research.

2. Target audience for web-based survey
Registered survey panel members

3. Use of results, protection of personal information
Your answers will be compiled into a statistical number, such as "XXX is the percentage of respondents who answered 'XXX'". Your name and personal information will not be disclosed. Your personal information will not be handled by the University of Yamanashi, which is the survey administrator. The results will never be used for any purpose other than research purposes.

Please read the above text carefully and select whether you are willing to participate in this survey or not.
1. Yes
2. No

[Q] In this question we will ask you about “research in which a human fertilized ovum (embryo) is cultivated outside the body beyond 14 days.”

［Video ⑧ Descriptions about research involving the culturing of human embryos in vitro beyond 14 days.］

Once a human fertilized ovum (embryo) passes the 14-day mark, the so-called “primitive streak” — which is a line that eventually becomes nerves — appears. Then, the organs and other structures subsequently form. Currently, the formation of the primitive streak is considered the point at which the fertilized ovum begins developing into a unique human being. After a cultivation experiment using such an ovum is completed, the fertilized ovum (embryo) is discarded; it is not transplanted into a human uterus.

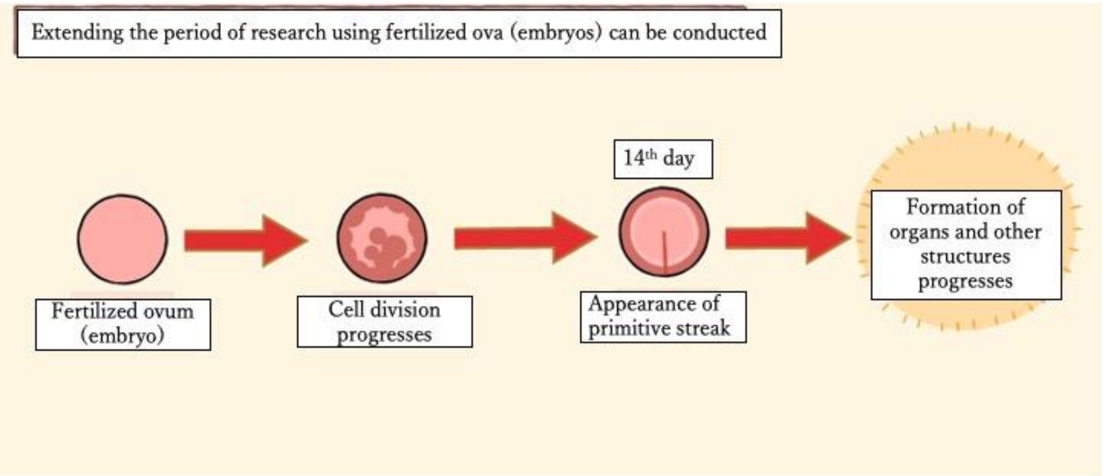

Do you think that research using fertilized ova (embryos) that are cultivated beyond 14 days should be allowed in Japan? [select one]

*Under current Japanese rules, human fertilized ova (embryos) can only be cultivated outside the body for a period of 14 days. However, in recent years there has been international debate over whether to abolish this rule.
*Anticipated outcome: Knowledge can be obtained that will be useful in the development of infertility treatments, the prevention of miscarriages, and the prevention of congenital diseases.
*Bioethical problem: Research using fertilized ova will be conducted for longer periods of time than currently allowed, and the fertilized ova used in this research will then be discarded.

1. It should be allowed
2. It should be prohibited
3. I cannot judge

(Branching question)

【The following question is for those who selected No. 2 (“It should be prohibited”)】

[Q] This question is for those who selected “It should be prohibited” in response to “cultivation for a period of more than 14 days.”
Do you think that the cultivation of human fertilized ova (embryos) for 14 days or less should be allowed? [select one]

1. It should be allowed
2. It should be prohibited
3. I cannot judge

[Q] Of the videos that you have seen…
［Video ①］ **Definition of stem cells**
［Video ②］ **Explanation of embryo models.**
［Video ③］ **Descriptions about research involving transplantation of human iPSCs into pig embryos to create pigs with organs derived from human iPSCs.**
［Video ④］ **Descriptions about research involving aborted fetal cells or tissue.**
…how well did you understand their contents? [select one]

1. I understood the contents
2. I understood the contents to some extent
3. I did not understand the contents very well
4. I did not understand the contents

[Q] Select the response that most accurately reflects your understanding of statements a through d below regarding regenerative medicine [select one per each]

	Correct	Incorrect	I do not know
a. Regenerative medicine requires the manipulation of human cells and the creation of organs and tissues	1	2	3
b. iPSCs are made from fertilized ova (embryos)	1	2	3
c. If human iPSCs are incorporated into fertilized pig ova (embryos), the human iPSCs die	1	2	3

[Q] Of the videos that you have seen …
［**Video** ⑤］ Explanations about in-vitro fertilization.
［**Video** ⑥］ Descriptions of research involving the fertilization of germ cells generated from iPSCs.
［**Video** ⑦］ Explanation on the use of mitochondrial replacement in human embryos for pregnancy.

［**Video** ⑧］ Descriptions about research involving the culturing of human embryos in vitro beyond 14 days.

…how well did you understand their contents? [select one]

1. I understood the contents

2. I understood the contents to some extent

3. I did not understand the contents very well

4. I did not understand the contents

[Q] Select the response that most accurately reflects your understanding of statements a through c below [select one per each]

	Correct	Incorrect	I do not know
a. It is impossible to fertilize an ovum with sperm outside the body.	1	2	3
b. The primitive streak appears around 14 days following the fertilization of an ovum (embryo) and is the basis for nerves.	1	2	3
c. In order to prevent a child from inheriting a disease originating in the mother's mitochondria, a method is used by which the ovum of a woman with altered mitochondria is replaced with the ovum of a woman with normal mitochondria.	1	2	3

[F] Do you currently have a religion? [select one]

1. No
2. Buddhist
3. Christian
4. Shinto
5. Islam
6. Other (please provide specifics:)
7. I don't want to answer

Table S1. Japanese guidelines in laboratory-based human embryo research under which the 14-day rule is defined

**The guidelines**	**Year of establishment (the latest revision)**	**Scope regarding "14-day rule"**
Guidelines for the Handling of Specified Embryos	2001 (2021)	Human cloned embryos, mitochondrial replacement of surplus human embryo.<br><br>Note: These guidelines were established under the Act on the Regulation of Human Cloning Techniques in 2000 (last revised in 2022).
Guidelines on the Derivation of Human ESCs	2014 (2022)	The generation of ESCs from surplus embryos.<br><br>Note: The Guidelines on the Utilization of Human ESCs in 2019 (last revised in 2022) define the procedures required for the use of ESCs.
Ethical Guidelines for Research that Involves the Use of Technology to Modify Genetic Information in Human Embryos	2019 (2022)	Genome editing on surplus embryos.
Ethical Guidelines for Assisted Reproductive Technology Research that Involves the Generation of Human Embryos	2010 (2022)	The creation of new embryos for the purpose of reproductive technology research.

Table S2. Intention to consider conducting research involving human embryo culture beyond 14 days among researchers who agreed with such research (n=247)

	n	%
Would consider	54	21.9
No plan to consider	158	64.0
Don’t know	35	14.2

Table S3. Relationship between research activities involving human embryos culture within 14 days and comprehension score and religious beliefs in the public

		Crude			Adjusted[a]		
	n	OR[b]	95% CI[c]	p-value	OR	95% CI	p-value
Agree (reference: cannot judge)							
Comprehension score[d]	276	2.56	1.76-3.72	<0.01	2.73	1.86-4.01	<0.01
Religion[e] (religious belief) reference: non-religious	263	1.50	0.83-2.71	0.18	1.53	0.85-2.76	0.16
Disagree (reference: cannot judge)							
Comprehension score	424	0.96	0.72-1.30	0.81	0.95	0.70-1.28	0.72
Religion (religious belief) reference: non-religious	393	2.44	1.45-4.13	<0.01	2.44	1.44-4.14	<0.01
Agree (reference: disagree)							
Comprehension score	452	2.65	1.91-3.66	<0.01	2.89	2.06-4.03	<0.01
Religion (religious belief) reference: non-religious	430	0.62	0.40-0.95	0.03	0.63	0.40-0.97	0.04

a Adjusted by age and sex

b OR=Odds Ratio

c 95% CI: 95% Confidential Interval

d Comprehension score was treated as 4 continuous scales, respectively, as shown in Table 1.

e Those who answered "I don’t want to answer" to the religion were excluded from the analysis.
