# Supplementary material for: Survey of Japanese researchers and the public regarding the culture of human embryos in vitro beyond 14 days
Source: Stem Cell Reports. 2023 Mar 23;18(4):799–806. doi: 10.1016/j.stemcr.2023.02.005 (PMC10147549; doi:10.1016/j.stemcr.2023.02.005)
Supplement: Document S2. Article plus supplemental information [file mmc2.pdf]

Stem Cell Reports
Report
OPEN ACCESS

Survey of Japanese researchers and the public regarding the culture of human embryos *in vitro* beyond 14 days

Hideki Yui,[1,*] Kaori Muto,[2] Yoshimi Yashiro,[3,4] Saori Watanabe,[2] Yukitaka Kiya,[2] Kumiko Fujisawa,[2] Kana Harada,[2] Yusuke Inoue,[2] and Zentaro Yamagata[1]
[1]University of Yamanashi Faculty of Medicine Graduate School of Medicine, Chuo City, Japan
[2]The University of Tokyo Institute of Medical Science, Minato-ku, Japan
[3]Tokyo Metropolitan Geriatric Hospital and Institute of Gerontology, Itabashi-ku, Japan
[4]Kanagawa University of Human Services School of Health Innovation, Kawasaki City, Japan
*Correspondence: hyui@yamanashi.ac.jp
https://doi.org/10.1016/j.stemcr.2023.02.005

SUMMARY

The International Society for Stem Cell Research (ISSCR) has eliminated its prohibition on research involving the culturing of human embryos beyond 14 days within the updated 2021 guidelines. We conducted a survey of Japanese researchers working in stem cell- or embryo-related research (n = 535) and the public (n = 3,000) about their attitudes toward the 14-day rule. Among the researchers, 46.2% agreed that embryos could be cultured beyond 14 days, a result that was slightly lower among the public (37.9%). Among those that disagreed with embryo culturing beyond 14 days, 9.5% of researchers and 5.1% of the public agreed with culturing embryos within 14 days. Among the public, higher comprehension levels correlated with both agreement and disagreement with the culture of embryos beyond 14 days compared with "cannot judge." Further research and pubic discourse are necessary in order to better understand the factors informing participant decisions regarding the 14-day rule.

INTRODUCTION

In 2021, the International Society for Stem Cell Research (ISSCR) updated their guidelines for stem cell research and clinical translation (ISSCR, 2021). In this update, the ISSCR eliminated the widely recognized prohibition involving the culturing of human embryos beyond 14 days. ISSCR recommend that if local policies and regulations permit, and if there is public support, specialized scientific and ethics oversight processes may permit research beyond 14 days (recommendation 2.2.2.1). The 14-day rule is reflected in numerous laws, regulations, and guidelines within many countries, where human embryos must be cultured *in vitro* for no longer than 14 days. ISSCR Guidelines Committee members explained the context in which these guidelines were developed (Lovell-Badge, 2021; Lovell-Badge et al., 2021; Anthony et al., 2021).

The 14-day rule has been adopted in Japan; however, the rules for human embryo research in Japan are complex (Yui et al., 2022). Table S1 highlights government guidelines that stipulate the 14-day rule. The Guidelines for the Handling of Specified Embryos are established under the Act on the Regulation of Human Cloning Techniques, while other guidelines established by the government are not linked to any law. Human cloned embryos, mitochondrial replacement on surplus embryos, derivation of human embryonic stem cells (ESCs) from surplus embryos, genome editing on surplus embryos, and creating new embryos for assisted reproductive technology research must be undertaken adhering to separate special guidelines. When initiating such research, scientists must undergo special ethics review as specified in the guidelines listed in Table S1. These guidelines stipulate that human embryos should only be used for research activities within 14 days after fertilization or before the appearance of the primitive streak. However, research using surplus embryos outside the scope of such guidelines are conducted in accordance with the guidelines for medical research involving human subjects (Ethical Guidelines for Medical and Biological Research Involving Human Subjects, established in 2021 and last revised in 2022). These guidelines are not specific to embryo research and thus do not contain any provisions regarding the 14-day rule. It is unlikely that the ethics review required under the guidelines would allow culture beyond 14 days. In addition, guidelines for gametogenesis from induced pluripotent stem cells (iPSCs) are defined in the Guidelines on the Research on Producing Germ Cells from Human iPSCs or Human Tissue Stem Cells (established in 2010 and last revised in 2022). However, since the creation of embryos using such germ cells is prohibited, the 14-day rule does not appear in these guidelines.

There have been several public and stakeholder perception studies that touch upon embryo research, including motivations among donors of human embryos. One systematic review identified that research purpose, treatment stage, embryo quality, religious beliefs, and altruism appeared to be important factors for donation

This is an open access article under the CC BY license (http://creativecommons.org/licenses/by/4.0/).

among embryo donors (Hug, 2008). In addition, several surveys of attitudes toward using human embryos in research, including ESCs, have been conducted (Hudson et al., 2005; Nisbet, 2005; Pardo and Calvo, 2008; Einsiedel et al., 2009; Evans and Kelley, 2011; Critchley et al., 2013). Regarding the extension of the 14-day rule, there were a total of 135 discussions on the topic in Chinese social media and news apps (Weibo, WeChat, and Tencent news) between May 27th and August 18, 2021, according to the Chinese big data public opinion platform Sina Yuqingtong, indicating that the overall attitude toward the debate was relatively neutral (46.21%) and supportive (31.82%) (Peng et al., 2022). However, there have been no large-scale surveys of stakeholder and public perceptions toward culturing embryos beyond 14 days in Japan or elsewhere. As the ISSCR guidelines stipulate the need for public support prior to culturing embryos beyond 14 days, it is critical to assess stakeholder and public attitudes toward embryo research that extends beyond 14 days. It is anticipated that such a policy shift would require greater discourse among academic societies and the public. Therefore, we conducted two surveys to assess the attitudes of stem cell and embryo researchers (members of the Japanese Society for Regenerative Medicine [JSRM] and those who were supported by the Japan Agency for Medical Research and Development [AMED], n = 535) and the public (n = 3,000) in Japan.

The detailed method of our surveys is described in Note S1. Further, a portion of the questionnaires translated into English (including the questions used in this manuscript) are available in Notes S2 and S3. Our surveys were conducted with the approval of the Institutional Review Board of the University of Yamanashi (approval number: CS0005).

RESULTS

Respondents' characteristics

Table 1 shows the characteristics of respondents. In the researcher group, there were 588 respondents, of whom 542 were members of the JSRM. We excluded 53 respondents, who did not engage in research activity, from the analysis. A total of 535 respondents met our inclusion criteria, and their responses were analyzed. Several members of the JSRM were conducting research with support from AMED, but we were unable to determine whether those from the AMED group who did not respond to the survey link were members of JSRM. Therefore, the exact response rate of researchers was unclear. However, among JSRM members, 8.96% responded to the survey.

Among those participating in the public survey, the age and sex ratios in the public group were consistent with the population distribution in Japan. Researchers were mostly in their 40s–50s, with fewer in their 20s, and more were male. Most participants reported as non-religious, followed by Buddhism, and a few reported non-Buddhist religions. The majority of participants (51.6%) had comprehension scores of 7–9 points, followed by 22.9% having a 4–6 comprehension score.

Attitudes toward the 14-day rule

When queried on the permissibility of culturing human embryos beyond 14 days under Japanese law and guidelines, 46.2% of researchers reported agreeing that human embryos should be cultured beyond 14 days, 29.3% reported being unable to judge, and 24.5% disagreed with the culturing of embryos beyond 14 days (Table 2). In comparison, 42.9% of the public reported that they cannot judge whether embryos should be cultured beyond 14 days, 37.9% agreed that human embryos should be cultured beyond 14 days, and 19.2% reported disagreeing with the culturing of embryos beyond 14 days. Significant differences were found in all responses between the two groups ($p < 0.01$).

Among participants in both groups who disagreed with culturing beyond 14 days, we asked about their level of agreement of culturing embryos within the 14-day limit. Of the 131 researchers who disagreed with culturing embryos beyond 14 days, 38.9% reported agreeing to culturing embryos within 14 days (9.5% of all researchers), and the same amount of researchers disagreed with culturing embryos within the 14-day limit (Table 2). Of the 576 members of the public who disagreed about embryo culturing beyond 14 days, 52.1% disagreed with culturing embryos within the 14-day limit (10% of all public participants), 26.4% (5.1% of all public participants) agreed to culturing within 14 days, and 21.5% (4.1% of all public participants) reported that they are unable to judge. Significant differences were found in "agree" and "disagree" between the two groups ($p < 0.01$ in both).

Researchers who agreed to culturing embryos beyond 14 days were asked whether they would consider conducting this research activity if the 14-day rule was abolished in Japan. Among the 247 researchers, 21.9% reported that they would consider conducting research where embryos were cultured beyond 14 days (10.1% of all researchers) (Table S2).

Relationship between research activities involving beyond/within 14 days of human embryo culture and comprehension level and religion in the public

Human embryo culture beyond 14 days

Members of the public with higher comprehension scores tended to agree that embryos should be cultured beyond 14 days when compared with those who reported being unable to judge; the difference was significant. For every one level increase in the comprehension score, the proportion of respondents who agreed increased (odds ratio

Table 1. Respondents' characteristics

	Researchers (n = 535)		The public (n = 3,000)	
	n	%	n	%
**Age**				
20–29	21	3.9	475	15.8
30–39	104	19.4	546	18.2
40–49	172	32.1	715	23.8
50–59	161	30.1	655	21.8
60–69	66	12.3	609	20.3
70 and over	11	2.1	–	–
**Sex**				
Male	413	77.2	1,517	50.6
Female	122	22.8	1,483	49.4
**Religion**				
Non-religious	–	–	1,998	66.6
Buddhism	–	–	674	22.5
Christianity	–	–	49	1.6
Shinto	–	–	74	2.5
Islam	–	–	3	0.1
Other	–	–	8	0.3
I don't want to answer	–	–	194	6.5
**Comprehension scores**				
0–3	–	–	262	8.7
4–6	–	–	686	22.9
7–9	–	–	1,548	51.6
10–12	–	–	504	16.8

[OR]: 2.62, 95% confidence interval [CI]: 2.34–2.94, $p < 0.01$) (Table 3). There was no significant difference among the groups with varying religious beliefs.

However, public members with higher comprehension scores also tended to disagree that embryos should be cultured beyond 14 days when compared with those who reported being unable to judge. For every one level increase in the comprehension score, the proportion of respondents who disagreed increased (OR: 2.18, 95% CI: 1.91–2.50, $p < 0.01$). There was no significant difference among the groups with varying religious orientations.

In the comparison between "agree" and "disagree," those with a higher comprehension score tended to "agree." For every one level increase in the comprehension score, the proportion of respondents who answered "agree" increased (OR: 1.20, 95% CI: 1.04–1.38, $p = 0.01$). There was no significant difference among the groups with varying religious orientations.

Human embryo culture within 14 days

Members of the public with higher comprehension scores tended to agree that the embryos should be cultured within 14 days when compared with those who reported being unable to judge; the difference was significant. For every one level increase in the comprehension score, the proportion of respondents who agreed increased (OR: 2.73, 95% CI: 1.86–4.01, $p < 0.01$) (Table S3). There was no significant difference among the groups with varying religious beliefs.

Public members with religious beliefs were more likely to disagree than those who were non-religious when compared with those who were unable to judge (OR: 2.44, 95% CI: 1.44–4.11, $p < 0.01$). There was no significant difference in the comprehension scores.

Table 2. Attitudes toward research activities involving human embryo cultures

Beyond 14 days

	Researchers (n = 535)		The public (n = 3,000)	
	n	%	n	%
Agree[a]	247	46.2	1,137	37.9
Cannot judge[a]	157	29.3	1,287	42.9
Disagree[a]	131	24.5	576	19.2

Within 14 days

	Researchers who disagreed with beyond 14 days (n = 131)		The public who disagreed with beyond 14 days (n = 576)	
	n	%	n	%
Agree[a]	51	38.9	152	26.4
Cannot judge	29	22.1	124	21.5
Disagree[a]	51	38.9	300	52.1

[a]$p < 0.01$. The chi-squared tests were performed for comparison between researchers and the public. p values were adjusted with the Bonferroni correction.

In the comparison between "agree" and "disagree," those with a higher comprehension score tended to "agree." For every one level increase in the comprehension score, the proportion of respondents who answered "agree" increased (OR: 2.89, 95% CI: 2.06–4.03, $p < 0.01$). In addition, those with religious beliefs tended not to agree when compared with those who were non-religious (OR: 0.63, 95% CI: 0.40–0.97, $p = 0.04$). In other words, non-religious public members were more likely to agree than those with religious beliefs.

Comparison of comprehension score among the public

We grouped the public based on their attitudes toward research involving human embryos. Table 4 presents the median and quartiles of the comprehension scores for each of the groups. [3-1] "disagree with beyond 14 days-agree with within 14 days" had the largest median and 25th and 75th percentile values, and [2] "cannot judge beyond 14 days" had the smallest. [1] "agree with beyond 14 days," [3-2] "disagree with beyond 14 days-cannot judge within 14 days" and [3-3] "disagree with beyond 14 days-disagree with within 14 days" had the same median and 75th percentile value; the 25th percentile value of [1] was higher than that of [3-2] and [3-3].

The score of [3-2] was the highest and that of [2] was the lowest, with significant differences when compared with all the other groups.

DISCUSSION

Analysis of results

Public perceptions of culturing embryos were somewhat similar to those of researchers, where the latter group were more agreeable to embryo culture beyond 14 days. At an initial glance, this observation may be due to researchers having greater insight on the value of conducting embryo research past 14 days, but nearly a quarter of the researchers still disagreed, and nearly a third were unable to judge the appropriateness of permitting embryo culture beyond 14 days. Our results also demonstrated that greater comprehension of stem cell and embryo research among members of the public indicated a greater likelihood of agreement with culturing embryos past day 14. However, even those who understood the content tended to disagree with the culture beyond 14 days. Therefore, a high level of comprehension does not lead to agreement with the culture beyond 14 days. This is exemplified by the fact that the highest comprehension scores were observed for participants who disagreed with the culture beyond 14 days while agreeing with the culture within 14 days. The percentage of such respondents was higher among researchers than among the public. These respondents could have found some valid reason for supporting the culture for the 14-day period. Therefore, even though the level of the public's comprehension can increase both the number of people in favor of and against it, it is important to provide knowledge for a sounder discussion.

Knowledge plays an important role in attitude determination. However, the simple deficit model, which posits that providing individuals with information will enhance their support for science and technology because a lack of knowledge is the root cause of their lack of support, has been met with criticism in the field of science communication (Stilgoe et al., 2014; Bauer, 2016). Attitude surveys on issues comparable to that in this study have revealed rejections of the deficit model. According to a survey conducted in Italy, a high level of scientific knowledge did not

Table 3. Relationship between research activities involving human embryos culture beyond 14 days and comprehension score and religious belief in the public

n		Crude			Adjusted[a]		
		OR	95% CI	p value	OR	95% CI	p value
**Agree (reference: cannot judge)**							
Comprehension score[b]	2,424	2.48	2.22–2.77	<0.01	2.62	2.34–2.94	<0.01
Religion[c] (religious beliefs) reference: non-religious	2,263	1.14	0.95–1.36	0.17	1.17	0.97–1.41	0.11
**Disagree (reference: cannot judge)**							
Comprehension score	1,863	2.18	1.91–2.49	<0.01	2.18	1.91–2.50	<0.01
Religion (religious beliefs) reference: non-religious	1,719	1.18	0.95–1.48	0.14	1.14	0.91–1.43	0.25
**Agree (reference: disagree)**							
Comprehension score	1,713	1.14	0.99–1.30	0.06	1.20	1.04–1.38	0.01
Religion (religious beliefs) reference: non-religious	1,630	0.96	0.77–1.20	0.72	1.02	0.81–1.29	0.85

OR, odds ratio. 95% CI, 95% confidence interval.
[a]Adjusted by age and sex.
[b]Comprehension score was treated as 4 continuous scales, respectively, as shown in Table 1.
[c]Those who answered "I don't want to answer" to the religion were excluded from the analysis.

necessarily result in a favorable attitude among the public toward biotechnology, such as genetic engineering of crops, the introduction of human genes into animals for organ transplants, research on human embryos, and reproductive cloning (Bucchi and Neresini, 2002). In addition, a survey of the Japanese public's and patients' attitudes toward germline genome editing showed that those with a higher level of comprehension tended to be more accepting of genome editing but were also more concerned about its risks (Uchiyama et al., 2018). These arguments are supported by our results.

The public was more likely to respond "cannot judge" to the culture beyond 14 days. This was not limited to research using human embryos but is characteristic of the Japanese attitude toward science and technology more generally. For example, in an international comparison of public attitudes toward whether regenerative medicine research should be promoted (Japan, South Korea, the US, the UK, Germany, and France), Japan had the highest percentage of respondents who answered "I don't know" (Shineha et al., 2022). Taking into account that the researchers were also Japanese, the fact that 29.3% of them answered "cannot judge" for culture beyond 14 days suggests that Japanese people may not want to cast a value judgment even if they have considerable knowledge and expertise in the subject area.

Inoue et al. surveyed the Japanese public and researchers about human-animal chimeric embryos for organ transplantation in 2012 and 2015 (Inoue et al., 2016). Their survey is comparable to the survey in this study because they surveyed members of the JSRM, which was the common population with this study; in addition, the answer choices were similar to those in this study. Among the researchers, 40.5% in 2012 (29.8% in 2015) responded "acceptable" and 31.3% (25.5%) responded "conditionally acceptable" to the creation of human-animal chimeric embryos, while 46.2% of researchers in our survey agree with culturing human embryos past day 14. Among the public, 7.5% in 2012 (6.4% in 2015) responded "acceptable" and 17.9% (16.2%) responded "conditionally acceptable" to the creation of human-animal chimeric embryos, while 37.9% of the public members in our survey agreed to culturing human embryos beyond 14 days. The comparison suggested that fewer respondents supported the creation of human-animal chimeric embryos than human embryo culturing beyond 14 days, among the public members. In addition, the higher acceptance of research activities by researchers than by the general public in Inoue et al.'s study was consistent with that in this study. The percentage of "cannot judge" was clearly higher for embryo cultures beyond 14 days for both researchers and the general public in this study than "undecided" for human-animal chimera in the Inoue et al. study. It was suggested that the intuitive value judgment for culturing human embryos beyond 14 days was more difficult than that for creating chimeric embryos and that the public intuitively fears chimeric animals. One possible context for this instinct is the potential for human-animal chimeras to create confusion in our current relationships with non-human animals and in our future relationships with such chimeras, as argued by Robert and Baylis (2003).

Religious beliefs did not affect the attitude toward culture beyond 14 days. This could be attributed to the

Table 4. Median and 25th and 75th percentiles of comprehension scores in each group and comparison between groups

	Median	25th and 75th percentiles	Comparison between groups[a]
[1] Agree with beyond 14 days	8	7 and 9	[1] and [2][b]<br>[1] and [3-1][b]<br>[1] and [3-2]<br>[1] and [3-3][b]
[2] Cannot judge beyond 14 days	7	4 and 8	[2] and [3-1][b]<br>[2] and [3-2][b]<br>[2] and [3-3][b]
[3-1] Disagree with beyond 14 days-agree with within 14 days	9	8 and 10	[3-1] and [3-2][b]<br>[3-1] and [3-3][b]
[3-2] Disagree with beyond 14 days-cannot judge within 14 days	8	6 and 9	[3-2] and [3-3]
[3-3] Disagree with beyond 14 days-disagree with within 14 days	8	6 and 9	–

[a]The Kruskal-Wallis test was performed and confirmed significant differences. The Dunn's test was then performed for comparison between groups, and p values were adjusted by the Bonferroni correction.
[b]$p < 0.01$.

situation in Japan, where the leaders of Buddhism, which constitutes the majority of religious belief, have not taken a particularly clear position on human embryo research or abortion. A survey of the Japanese public's attitude toward the process of creating germ cells from iPSCs and using them to create embryos also found no relationship between religious beliefs and attitude (Sawai et al., 2021). In interpreting these results, it is necessary to bear in mind that most Japanese people are not avidly religious. There are many Japanese people whose association with Buddhism is limited to special circumstances, i.e., calling a priest at funerals, and some Japanese people may identify as either "Buddhist" or "non-religious." Therefore, a more definitive result could have been obtained if the study was based on the strength of faith or religiosity. In addition, in the Japanese context, even if a person self-identifies as non-religious, there are many cases where there is no strong belief but merely a lack of interest and so a simple comparison with the non-religious stance in the Western world is not possible. Since 95% of the respondents were either non-religious or Buddhist, identifying differences based on religiosity would not be possible as it would require a larger sample size.

Considering the diversity in responses from our survey among members of the public and researchers, greater dialogue is needed not only between researchers and the public but also among researchers. Our findings serve as a preliminary set of results about public and stakeholder attitudes toward the 14-day rule. Yet, further research is needed to deepen the factors behind the various decisions or why some participants feel they cannot make a decision on the permissibility of culturing embryos past day 14.

Limitations of the study

Several limitations of our study are worth noting. Firstly, there could be sampling bias in recruitment of participants who are technologically savvy and would participate in web-based surveys. Therefore, we did not include those who were over 69 years in the survey for the public. Moreover, very few young researchers responded to the survey, and the response rate of researchers was low. In addition, it remains unclear how respondents' attitudes may have been impacted by the explanations and information provided in the videos. We did not verify, through cognitive interviews or other means, how participants understood the content of the questions. Furthermore, another study from our group indicates that the explanatory video used in this survey improved public understanding; however, that manuscript has not yet passed peer review. Therefore, it cannot be ruled out that the information provided could have biased participant responses. However, it is important to note that the large number of "cannot judge" responses from the general public is consistent with the characteristics of Japanese public attitudes toward science and technology, a finding that can contribute to policymaking.

Conclusion

This study is one of the first large-scale surveys of researcher and public attitudes toward research activities involving the culture of human embryos beyond 14 days and serves as a reference for future policy decisions. It is anticipated

that there will be policy debate regarding the length of time that embryos can be cultured in the near future. This survey indicates that not many people disagree with the elimination of the 14-day rule. However, those who do agree are less than 50% among both researchers and the public, at least in the Japanese context. It is unlikely that many members of the public will be able to judge the pros and cons of such a debate. To raise the level of the debate, information dissemination will be necessary so that the public is well informed of the pros and cons of expanding or eliminating the 14-day rule. It is imperative that future research and dialogue be conducted among key stakeholder groups and the public to help inform policy decisions.

EXPERIMENTAL PROCEDURES

Resource availability

Corresponding author

Further information and requests for resources should be directed to and will be fulfilled by the corresponding author, Hideki Yui (hyui@yamanashi.ac.jp).

Materials availability

This study did not generate new unique reagents.

Data and code availability

A minimal dataset of the researchers is accessible through https://figshare.com/articles/dataset/Dataset_the_researchers_/20417946.

A minimal dataset of the public is accessible through https://figshare.com/articles/dataset/Dataset_the_public_/20417907.

Statistical analysis

Chi-squared tests were performed to compare the responses of the researchers and the public; p values were adjusted using the Bonferroni correction (Table 2). Multinominal logistic regression analyses were performed to examine the association of the attitudes toward research involving human embryo cultures and comprehension level and religion in the public (Tables 3 and S3). The Kruskal-Wallis test followed by the Dunn's test were used to compare the comprehension scores of the groups of the public classified by different attitudes toward embryo research; p values were adjusted using the Bonferroni correction (Table 4).

The significance level was set to 0.05 (5%) in each analysis. Data were analyzed using IBM-Statistical Package for the Social Sciences (v.27). Detail information is available in Note S1.

SUPPLEMENTAL INFORMATION

Supplemental information can be found online at https://doi.org/10.1016/j.stemcr.2023.02.005.

AUTHOR CONTRIBUTIONS

Conceptualization, H.Y., K.M., and Z.Y.; data curation, H.Y., S.W., and K.F.; formal analysis, H.Y.; investigation, H.Y., K.M., Y.Y., S.W., Y.K., K.F., and Z.Y.; methodology, H.Y., K.M., and Z.Y.; writing – original draft, H.Y.; writing – review & editing, K.M., Y.Y., S.W., Y.K., K.F., K.H., Y.I., and Z.Y.; project administration, K.M.; funding acquisition, Y.Y. and Z.Y.; validation, K.H., Y.I., and Z.Y.; supervision, Z.Y.

ACKNOWLEDGMENTS

This research was supported by AMED under grant number JP 20bm0904002.

CONFLICT OF INTERESTS

The authors declare no competing interests.

Received: August 15, 2022
Revised: February 17, 2023
Accepted: February 18, 2023
Published: March 23, 2023

REFERENCES

Anthony, E., Lovell-Badge, R., and Morrison, S.J. (2021). New guidelines for stem cell and embryo research from the ISSCR. Cell Stem Cell *28*, 991–992. https://doi.org/10.1016/j.stem.2021.05.009.

Bauer, M.W. (2016). Results of the essay competition on the 'deficit concept. Publ. Understand. Sci. *25*, 398–399. https://doi.org/10.1177/0963662516640650.

Bucchi, M., and Neresini, F. (2002). Biotech remains unloved by the more informed. Nature *416*, 261. https://doi.org/10.1038/416261a.

Critchley, C.R., Bruce, G., and Farrugia, M. (2013). The impact of commercialisation on public perceptions of stem cell research: exploring differences across the use of induced pluripotent cells, human and animal embryos. Stem Cell Rev. Rep. *9*, 541–554. https://doi.org/10.1007/s12015-013-9445-4.

Einsiedel, E., Premji, S., Geransar, R., Orton, N.C., Thavaratnam, T., and Bennett, L.K. (2009). Diversity in public views toward stem cell sources and policies. Stem Cell Rev. Rep. *5*, 102–107. https://doi.org/10.1007/s12015-009-9063-3.

Evans, M.D.R., and Kelley, J. (2011). US attitudes toward human embryonic stem cell research. Nat. Biotechnol. *29*, 484–488. https://doi.org/10.1038/nbt.1891.

Hudson, K., Scott, J., and Faden, R. (2005). Values in Conflict: Public Attitudes on Embryonic Stem Cell Research (Genetics and Public Policy Center).

Hug, K. (2008). Motivation to donate or not donate surplus embryos for stem-cell research: literature review. Fertil. Steril. *89*, 263–277. https://doi.org/10.1016/j.fertnstert.2007.09.017.

Inoue, Y., Shineha, R., and Yashiro, Y. (2016). Current public support for human-animal chimera research in Japan is limited, despite high levels of scientific approval. Cell Stem Cell *19*, 152–153. https://doi.org/10.1016/j.stem.2016.07.011.

ISSCR (2021). ISSCR Guidelines for Stem Cell Research and Clinical Translation. https://www.isscr.org/docs/default-source/all-isscr-guidelines/2021-guidelines/isscr-guidelines-for-stem-cell-research-and-clinical-translation-2021.pdf?sfvrsn=979d58b1_4.

Lovell-Badge, R. (2021). Stem-cell guidelines: why it was time for an update. Nature *593*, 479. https://doi.org/10.1038/d41586-021-01387-z.

Lovell-Badge, R., Anthony, E., Barker, R.A., Bubela, T., Brivanlou, A.H., Carpenter, M., Charo, R.A., Clark, A., Clayton, E., Cong, Y., et al. (2021). ISSCR guidelines for stem cell research and clinical translation: the 2021 update. Stem Cell Rep. *16*, 1398–1408. https://doi.org/10.1016/j.stemcr.2021.05.012.

Nisbet, M.C. (2005). The competition for worldviews: values, information, and public support for stem cell research. Int. J. Publ. Opin. Res. *17*, 90–112. https://doi.org/10.1093/ijpor/edh058.

Pardo, R., and Calvo, F. (2008). Attitudes toward embryo research, worldviews, and the moral status of the embryo frame. Sci. Commun. *30*, 8–47. https://doi.org/10.1177/1075547008319432.

Peng, Y., Lv, J., Xiao, Z., Ding, L., and Zhou, Q. (2022). A framework for the responsible reform of the 14-day rule in human embryo research. Protein Cell *13*, 552–558. https://doi.org/10.1007/s13238-022-00907-5.

Robert, J.S., and Baylis, F. (2003). Crossing species boundaries. Am. J. Bioeth. *3*, 1–13. https://doi.org/10.1162/15265160360706417.

Sawai, T., Hatta, T., Akatsuka, K., and Fujita, M. (2021). Public attitudes in Japan toward the creation and use of gametes derived from human-induced pluripotent stem cells. Future Sci. OA *7*, FSO755. https://doi.org/10.2144/fsoa-2021-0066.

Shineha, R., Inoue, Y., and Yashiro, Y. (2022). A comparative analysis of attitudes toward stem cell research and regenerative medicine between six countries - a pilot study. Regen. Ther. *20*, 187–193. https://doi.org/10.1016/j.reth.2022.04.007.

Stilgoe, J., Lock, S.J., and Wilsdon, J. (2014). Why should we promote public engagement with science? Publ. Understand. Sci. *23*, 4–15. https://doi.org/10.1177/0963662513518154.

Uchiyama, M., Nagai, A., and Muto, K. (2018). Survey on the perception of germline genome editing among the general public in Japan. J. Hum. Genet. *63*, 745–748. https://doi.org/10.1038/s10038-018-0430-2.

Yui, H., Muto, K., Yashiro, Y., Watanabe, S., Kiya, Y., Kamisato, A., Inoue, Y., and Yamagata, Z. (2022). Comparison of the 2021 International Society for Stem Cell Research (ISSCR) guidelines for "laboratory-based human stem cell research, embryo research, and related research activities" and the corresponding Japanese regulations. Regen. Ther. *21*, 46–51. https://doi.org/10.1016/j.reth.2022.05.002.

Stem Cell Reports, Volume *18*

Supplemental Information

Survey of Japanese researchers and the public regarding the culture of human embryos *in vitro* beyond 14 days

Hideki Yui, Kaori Muto, Yoshimi Yashiro, Saori Watanabe, Yukitaka Kiya, Kumiko Fujisawa, Kana Harada, Yusuke Inoue, and Zentaro Yamagata

Note S1. Method of the surveys

1 Research participants and data collection

We conducted two web-based attitude surveys involving a specific group of researchers and the public. As web-based surveys, informed consent was obtained from all participants by requesting them to click on a checkbox within the online platform.

1.1 Researchers

A link to the survey screen was sent to all members of the Japanese Society for Regenerative Medicine (JSRM) via email and mail at the same time. A follow-up email was sent after two weeks. In total, two emails and one mail were sent by JSRM. The same link was included in all correspondence to participants. JSRM is a national scientific society with 6052 members, most of whom are biomedical researchers working in stem cells and regenerative medicine. Researchers who are members of JSRM are likely to constitute the bulk of Japanese biomedical researchers and thus serves as a convenience sample of biomedical researchers knowledgeable in regenerative medicine research. The Japan Agency for Medical Research and Development (AMED) is a government funding agency dedicated to funding ~~bio~~medical research. A total of 345 researchers, who were supported by the Project for Regenerative/Cellular Medicine and Gene Therapies were emailed. Follow-up emails were sent after two and three weeks. In total, three emails were sent by AMED. The survey remained open from March 2 to 31, 2022.

JSRM members included non-researchers such as the journalists. We excluded those who answered that they did not engage in research activities, from the analysis because our goal was to analyze the views of biomedical researchers engaged in stem cell and regenerative medicine research.

1.2 The public

To survey the public, participants were recruited from those who had voluntarily registered with a panel of the Nippon Research Center (https://www.nrc.co.jp/english/index.html). Nippon Research Center is a private company that conducts marketing and public opinion studies on a contractual basis. We commissioned the Nippon Research Center to recruit participants, host the online survey, and collect participant responses. Adults (aged 20−69 years) were recruited randomly until the target number of 3000 respondents was reached. At the time of the survey, the age of adulthood in Japan was 20 years old. According to the Japan Research Center (in response to our inquiry), the age distribution of the panel members was as follows: 7.4% were 15−19 years old, 19.5% were in their 20s, 21.9% in their 30s, 22.2% in their 40s, 18.9% in their 50s, 7.7% in their 60s, 2.1% in their 70s, 0.3 % in their 80s, and 0.02% in their 90s. Among Japan's

population of those over 14 years old in 2022, 14.2% were in their 70s, 8.4% were in their 80s, and 2.2% were in their 90s (Statistics Bureau of Japan, 2023). The proportion of those over the age of 69 enrolled in the panel was small compared to the distribution of the overall Japanese population. One reason for the gap was that most members of the panel regularly used the internet, unlike the majority of the elderly population. Therefore, there was a potential for bias as those over the age of 69 enrolled in the panel were a distinct group within their generation. Thus, the upper age limit for respondents was set at 69 years-old. Data collection was conducted from January 5 to 13, 2022 until the number of participants reached 3,000.

2 Survey items

The survey asked for opinions on several stem cell- and embryo-related research activities and was part of a larger survey study which captured stakeholder and public views on creating embryo using gamete generated from iPSCs, using such embryos for pregnancy, creating embryo models, creating a human-pig chimera, using organs from human-pig chimeras for transplantation into a patient, and transferring an embryo after mitochondrial replacement therapy into a woman for pregnancy, and using human fetal tissue for medical research. The surveys were conducted in Japanese. The questionnaires were developed by our research team, which included experts in stem cell science and prior experience in conducting perception surveys. Survey questions were shown on individual screens and once a participant responded to the question, they were unable to return to the previous screen. Nippon Research Center's online survey platform was used to host both web-based surveys. The questions used in this study were primarily related to research activities involving the culture of human embryos beyond 14 days. Background information on these research activities, including the status of domestic regulations, were also presented.

2.1 Questions for the researchers

Attitudes about the culture of human embryos beyond 14 days were phrased as "Should research in which human embryos are cultured beyond 14 days be allowed under Japanese law and guidelines, when the embryos are not used for pregnancy?" The potential fixed responses included: "should be allowed," "cannot judge," and "should be prohibited." For respondents who answered that embryo culture beyond 14 days "should be prohibited," a subsequent question was posed: "Should embryo culture that is within 14 days also be prohibited?" and were permitted similar response categories ("should be allowed," "cannot judge," "should be prohibited"). For respondents who answered "should be allowed" were asked about a follow-up question whether they would consider conducting embryo research beyond 14 days if the 14-day rule was abolished in Japan. The responses included: "would consider," "no plan to consider," and "don't know."

2.2 Questions for the public

Members of the public were asked similar sets of questions to researchers. An initial question, “Do you think that research using fertilized ova (embryos) that are cultured beyond 14 days should be allowed in Japan?” with potential fixed responses included: “should be allowed,” “cannot judge,” and “should be prohibited.” For those respondents who answered “should be prohibited,” a follow-up question (“Do you think that the culture of human fertilized ova (embryos) within 14 days should be allowed?”) was posed with the same fixed response categories.

We used a newly developed video to provide participants a backgrounder on embryo research (video available from: https://figshare.com/articles/media/Explanation_by_video/19977308). The video comprised two separate sections with eight parts. The usefulness of the video at improving the public’s understanding of embryo research was confirmed in a separate study (manuscript currently under review). We scored content comprehension based on six correct/incorrect questions regarding the video contents (see Note S3) and subjective comprehension of the videos. For each correct answer, one point was added. To rate their subjective comprehension of the video, we asked respondents “How well did you understand the contents of video?” The answer choices were “I understood the contents” (three points), “I understood the contents to some extent” (two points), “I did not understand the contents very well” (one point) and “I did not understand the contents” (zero points). Subjective comprehension was evaluated for each two parts of the video. The maximum comprehension score was 12 points. In this point system, a higher score indicated a better understanding.

3 Analysis method

A simple tabulation of the results of attitudes from researchers and the public toward the culture of human embryos beyond/within 14 days was presented. Chi-square tests were performed to compare the responses of the researchers and the public; P-values were adjusted using the Bonferroni correction.

We examined the association of the attitudes toward research involving human embryos and comprehension level in the public. We performed a multinomial logistic regression analysis with attitudes toward embryo culture beyond/within 14 days as the dependent variable, while adjusting for age and sex as covariates. Additionally, to consider the influence of religion, the same analysis was undertaken between two groups: religious beliefs and non-religious. Age and comprehension scores were considered on 5 and 4 continuous scales, respectively (Table 1).

We grouped the respondents in the public based on their attitudes toward research using human embryos, and compared their comprehension scores. The grouping was as follows:

[1] “agree with beyond 14 days,” [2] “cannot judge beyond 14 days,” [3-1] “disagree with beyond 14 days – agree with within 14 days,” [3-2] “disagree with beyond 14 days – cannot judge within 14 days,” and [3-3] “disagree with beyond 14 days – disagree with within 14 days.” Median and quartile of comprehension scores for each group were calculated. Kruskal-Wallis test followed by a Dunn’s test was used for comparison between groups; P values were adjusted using Bonferroni correction.

The significance level was set to 0.05 (5%) in each analysis. Data were analyzed using IBM-Statistical Package for the Social Sciences (version 27).

Reference

Statistics Bureau of Japan. (2022). Population Estimates: January 2023 Report (in Japanese). https://www.stat.go.jp/data/jinsui/pdf/202301.pdf.

Note S2. Questionary for the researchers

Survey of Attitudes in Researchers About Stem Cell or Embryo-Related Research

Request for responses to our web-based survey

In May 2021, the International Society for Stem Cell Research (ISSCR) published new guidelines. The guidelines specify what research is acceptable and what is not acceptable in stem cell- and embryo-related research.
The purpose of this survey is to gather the opinions of researchers conducting research on stem cells and regenerative medicine in order to consider the future of Japanese laws, regulations, and guidelines for related research. In this survey, we ask for your opinions on several research activities (e.g., research in which human embryos are cultured beyond 14 days, research to create embryo models, etc.) in light of the ISSCR guidelines and Japanese laws, regulations, and guidelines.

We invite you this survey through the Japan Society for Regenerative Medicine (JSRM) and Japan Agency for Medical Research and Development (AMED). Therefore, some respondents may have received duplicate invitations to this survey. The survey can be accessed via the QR code on the mail from JSRM or the URL on the e-mail sent by JSRM or AMED. You may access the survey from any location, but we ask you to answer the survey only once per person.

1. The purpose of the web-based survey
We ask for the public's thoughts on ethical and social issues regarding regenerative medicine and stem cell research.

2. Target audience for web-based survey
Members of JSRM. Researchers conducting research related to stem cells or embryos supported by AMED.

3. Use of results, protection of personal information
Your answers will be compiled into a statistical number, such as "XXX is the percentage of respondents who answered 'XXX'". Your name and personal information will not be disclosed. Your personal information will not be handled by the University of Tokyo, University of Yamanashi, Tokyo Metropolitan Geriatric Hospital and Institute of Gerontology which are the survey

administrators. The results will never be used for any purpose other than research purposes.

Please read the above text carefully and select whether you are willing to participate in this survey or not.

1. Yes
2. No

[F] What is your age? [select one]

1. 0 – 19 years
2. 20 – 29 years
3. 30 – 39 years
4. 40 – 49 years
5. 50 – 59 years
6. 60 – 69 years
7. 70 years and above

[F] What is your sex? [select one]

1. Female
2. Male

[F] Did you access this survey screen via email or mail from JSRM or E from AMED?

1. E-mail or mail from JSRM

2. E-mail from AMED

[F] For those who accessed via e-mail from AMED. Are you a member of JSRM?

1. Yes

2. No

[Q] Should research in which human embryos are cultured beyond 14 days be allowed under Japanese law and guidelines, when the embryos are not used for pregnancy?

The ISSCR Guidelines (until the 2016 edition) prohibited the culture of embryos beyond 14 days, but the 2021 revision removed this prohibition. The 2021 guidelines state that the culture of embryos beyond 14 days may be performed after specialized ethical review.

In Japan, the relevant guidelines (*) prohibit culture beyond 14 days, on the grounds that the

primitive streak appears, and body organs begin to form by this time.

1. It should be allowed
2. It should be prohibited
3. I cannot judge

(*) Guidelines for the Handling of Specified Embryos, Guidelines on the Derivation of Human ESCs, Ethical Guidelines for Research that Involves the Use of Technology to Modify Genetic Information in Human Embryos, and Ethical Guidelines for Assisted Reproductive Technology Research that Involves the Generation of Human Embryos.

[Q] For those who answered “It should be prohibited” in embryo culture beyond 14 days. Should the embryo culture that is within 14 days also be prohibited?

1. It should be allowed
2. It should be prohibited
3. I cannot judge

[Q] For those who answered “It should be allowed” in embryo culture beyond 14 days. If Japanese laws, regulations, and guidelines were to allow this research in the future, would you consider conducting this research?

1. I would consider.
2. I have no plan to consider.
3. I don’t know.

Note S3. Questionary for the public

Survey of Public Attitudes About Stem Cell or Embryo-Related Research

[SC] What is your sex? [select one]

1. Female
2. Male

[SC] What is your age? [select one]

1. 0 – 19 years
2. 20 – 24 years
3. 25 – 29 years
4. 30 – 34 years
5. 35 – 39 years
6. 40 – 44 years
7. 45 – 49 years
8. 50 –- 54 years
9. 55 – 59 years
10. 60 – 64 years
11. 65 – 69 years
12. 70 years and above

Request for responses to our web-based survey

In recent years, the state of medical research involving human subjects has been changing rapidly, with particularly remarkable progress in regenerative medicine and stem cell research.

Regenerative medicine" is a medical treatment that aims to artificially manipulate the regenerative ability of tissues to restore impaired tissues and organs to their normal state. Experiments are being conducted to process human cells and fertilized ovum (embryo) in order to realize regenerative medicine. However, because of various bioethical concerns, there is an ongoing effort to promote international alignment on what types of experiments are acceptable. In addition to researchers, the opinions of patients, their families, and the general public are also important. This survey asks about the public's awareness of the state of regulation of research on human stem cells and fertilized eggs (embryos). We apologize for the inconvenience this may

cause you, but we appreciate your cooperation.

1. The purpose of the web-based survey
We ask for the public's thoughts on ethical and social issues regarding regenerative medicine and stem cell research.

2. Target audience for web-based survey
Registered survey panel members

3. Use of results, protection of personal information
Your answers will be compiled into a statistical number, such as "XXX is the percentage of respondents who answered 'XXX'". Your name and personal information will not be disclosed. Your personal information will not be handled by the University of Yamanashi, which is the survey administrator. The results will never be used for any purpose other than research purposes.

Please read the above text carefully and select whether you are willing to participate in this survey or not.
1. Yes
2. No

[Q] In this question we will ask you about “research in which a human fertilized ovum (embryo) is cultivated outside the body beyond 14 days.”

〔Video ⑧ Descriptions about research involving the culturing of human embryos in vitro beyond 14 days.〕

Once a human fertilized ovum (embryo) passes the 14-day mark, the so-called “primitive streak” — which is a line that eventually becomes nerves — appears. Then, the organs and other structures subsequently form. Currently, the formation of the primitive streak is considered the point at which the fertilized ovum begins developing into a unique human being. After a cultivation experiment using such an ovum is completed, the fertilized ovum (embryo) is discarded; it is not transplanted into a human uterus.

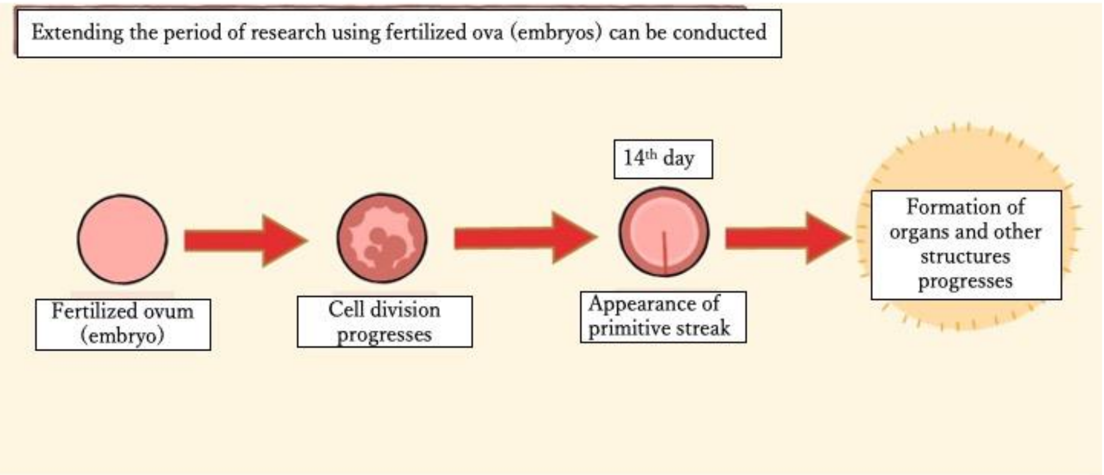

Do you think that research using fertilized ova (embryos) that are cultivated beyond 14 days should be allowed in Japan? [select one]

*Under current Japanese rules, human fertilized ova (embryos) can only be cultivated outside the body for a period of 14 days. However, in recent years there has been international debate over whether to abolish this rule.
*Anticipated outcome: Knowledge can be obtained that will be useful in the development of infertility treatments, the prevention of miscarriages, and the prevention of congenital diseases.
*Bioethical problem: Research using fertilized ova will be conducted for longer periods of time than currently allowed, and the fertilized ova used in this research will then be discarded.

1. It should be allowed
2. It should be prohibited
3. I cannot judge

(Branching question)

【The following question is for those who selected No. 2 (“It should be prohibited”)】

[Q] This question is for those who selected “It should be prohibited” in response to “cultivation for a period of more than 14 days.”
Do you think that the cultivation of human fertilized ova (embryos) for 14 days or less should be allowed? [select one]

1. It should be allowed
2. It should be prohibited
3. I cannot judge

[Q] Of the videos that you have seen…

［Video ①］ **Definition of stem cells**

［Video ②］ **Explanation of embryo models.**

［Video ③］ **Descriptions about research involving transplantation of human iPSCs into pig embryos to create pigs with organs derived from human iPSCs.**

［Video ④］ **Descriptions about research involving aborted fetal cells or tissue.**

…how well did you understand their contents? [select one]

1. I understood the contents
2. I understood the contents to some extent
3. I did not understand the contents very well
4. I did not understand the contents

[Q] Select the response that most accurately reflects your understanding of statements a through d below regarding regenerative medicine [select one per each]

	Correct	Incorrect	I do not know
a. Regenerative medicine requires the manipulation of human cells and the creation of organs and tissues	1	2	3
b. iPSCs are made from fertilized ova (embryos)	1	2	3
c. If human iPSCs are incorporated into fertilized pig ova (embryos), the human iPSCs die	1	2	3

[Q] Of the videos that you have seen …

［**Video** ⑤］ Explanations about in-vitro fertilization.

［**Video** ⑥］ Descriptions of research involving the fertilization of germ cells generated from iPSCs.

［**Video** ⑦］ Explanation on the use of mitochondrial replacement in human embryos for pregnancy.

［**Video** ⑧］ Descriptions about research involving the culturing of human embryos in vitro beyond 14 days.

…how well did you understand their contents? [select one]

1. I understood the contents

2. I understood the contents to some extent

3. I did not understand the contents very well

4. I did not understand the contents

[Q] Select the response that most accurately reflects your understanding of statements a through c below [select one per each]

	Correct	Incorrect	I do not know
a. It is impossible to fertilize an ovum with sperm outside the body.	1	2	3
b. The primitive streak appears around 14 days following the fertilization of an ovum (embryo) and is the basis for nerves.	1	2	3
c. In order to prevent a child from inheriting a disease originating in the mother's mitochondria, a method is used by which the ovum of a woman with altered mitochondria is replaced with the ovum of a woman with normal mitochondria.	1	2	3

[F] Do you currently have a religion? [select one]

1. No
2. Buddhist
3. Christian
4. Shinto
5. Islam
6. Other (please provide specifics:)
7. I don't want to answer

Table S1. Japanese guidelines in laboratory-based human embryo research under which the 14-day rule is defined

The guidelines	Year of establishment (the latest revision)	Scope regarding "14-day rule"
Guidelines for the Handling of Specified Embryos	2001 (2021)	Human cloned embryos, mitochondrial replacement of surplus human embryo.<br><br>Note: These guidelines were established under the Act on the Regulation of Human Cloning Techniques in 2000 (last revised in 2022).
Guidelines on the Derivation of Human ESCs	2014 (2022)	The generation of ESCs from surplus embryos.<br><br>Note: The Guidelines on the Utilization of Human ESCs in 2019 (last revised in 2022) define the procedures required for the use of ESCs.
Ethical Guidelines for Research that Involves the Use of Technology to Modify Genetic Information in Human Embryos	2019 (2022)	Genome editing on surplus embryos.
Ethical Guidelines for Assisted Reproductive Technology Research that Involves the Generation of Human Embryos	2010 (2022)	The creation of new embryos for the purpose of reproductive technology research.

Table S2. Intention to consider conducting research involving human embryo culture beyond 14 days among researchers who agreed with such research (n=247)

	n	%
Would consider	54	21.9
No plan to consider	158	64.0
Don't know	35	14.2

Table S3. Relationship between research activities involving human embryos culture within 14 days and comprehension score and religious beliefs in the public

		Crude			Adjusted[a]		
	n	OR[b]	95% CI[c]	p-value	OR	95% CI	p-value
Agree (reference: cannot judge)							
Comprehension score[d]	276	2.56	1.76-3.72	<0.01	2.73	1.86-4.01	<0.01
Religion[e] (religious belief) reference: non-religious	263	1.50	0.83-2.71	0.18	1.53	0.85-2.76	0.16
Disagree (reference: cannot judge)							
Comprehension score	424	0.96	0.72-1.30	0.81	0.95	0.70-1.28	0.72
Religion (religious belief) reference: non-religious	393	2.44	1.45-4.13	<0.01	2.44	1.44-4.14	<0.01
Agree (reference: disagree)							
Comprehension score	452	2.65	1.91-3.66	<0.01	2.89	2.06-4.03	<0.01
Religion (religious belief) reference: non-religious	430	0.62	0.40-0.95	0.03	0.63	0.40-0.97	0.04

a Adjusted by age and sex

b OR=Odds Ratio

c 95% CI: 95% Confidential Interval

d Comprehension score was treated as 4 continuous scales, respectively, as shown in Table 1.

e Those who answered "I don't want to answer" to the religion were excluded from the analysis.
